# Innate Invariant NKT Cells Recognize *Mycobacterium tuberculosis–*Infected Macrophages, Produce Interferon-γ, and Kill Intracellular Bacteria

**DOI:** 10.1371/journal.ppat.1000239

**Published:** 2008-12-12

**Authors:** Isabel Sada-Ovalle, Asako Chiba, Adaena Gonzales, Michael B. Brenner, Samuel M. Behar

**Affiliations:** 1 Division of Rheumatology, Immunology, and Allergy, Brigham and Women's Hospital and Harvard Medical School, Boston, Massachusetts, United States of America; 2 Immunochemistry Department, National Institute of Respiratory Diseases, Mexico City, Mexico; University of Pittsburgh School of Medicine, United States of America

## Abstract

Cellular immunity to *Mycobacterium tuberculosis* (*Mtb*) requires a coordinated response between the innate and adaptive arms of the immune system, resulting in a type 1 cytokine response, which is associated with control of infection. The contribution of innate lymphocytes to immunity against *Mtb* remains controversial. We established an in vitro system to study this question. Interferon-γ is produced when splenocytes from uninfected mice are cultured with *Mtb*-infected macrophages, and, under these conditions, bacterial replication is suppressed. This innate control of bacterial replication is dependent on CD1d-restricted invariant NKT (iNKT) cells, and their activation requires CD1d expression by infected macrophages as well as IL-12 and IL-18. We show that iNKT cells, even in limiting quantities, are sufficient to restrict *Mtb* replication. To determine whether iNKT cells contribute to host defense against tuberculosis in vivo, we adoptively transferred iNKT cells into mice. Primary splenic iNKT cells obtained from uninfected mice significantly reduce the bacterial burden in the lungs of mice infected with virulent *Mtb* by the aerosol route. Thus, iNKT cells have a direct bactericidal effect, even in the absence of synthetic ligands such as α-galactosylceramide. Our finding that iNKT cells protect mice against aerosol *Mtb* infection is the first evidence that CD1d-restricted NKT cells mediate protection against *Mtb* in vivo.

## Author Summary

Host resistance to *Mycobacterium tuberculosis* (*Mtb*) requires a coordinated response by the different components of the immune system. We established an in vitro model to study the contribution of innate lymphocytes to immunity against *Mtb*. When co-cultured with *Mtb*-infected macrophages, splenocytes from uninfected mice become activated and suppress bacterial replication. By fractionating the different splenocyte cell populations, we discovered that the invariant NKT (iNKT) cell is essential for suppressing intracellular bacterial replication. iNKT cells, which are conserved in rodents and humans, recognize lipids presented by the antigen-presenting molecule CD1d. While we had previously shown that iNKT cell-deficient mice are not more susceptible to tuberculosis, a potential contribution of iNKT cells during the early phase of immunity may have been masked. To address this issue, we showed that highly purified iNKT cells were sufficient to reduce the lung bacterial burden of mice infected with virulent *Mtb*. This is the first evidence that CD1d-restricted iNKT cells play a physiological role in mediating protection against aerosol *Mtb* infection in vivo. Thus, by being an early producer of interferon-g and suppressing intracellular bacterial growth, iNKT cells function as an important part of the early immune response against *Mtb*.

## Introduction

Cells of the innate immune system use several receptor systems to recognize pathogens and act as the first line of defense against infection. In contrast, the expression of clonal antigen receptors and the capacity to differentiate into memory cells distinguish B and T lymphocytes as the central components of the adaptive immune system. Certain T subsets, such as γδ T cells and NKT cells, have features of innate immune cells including a partially activated phenotype, a rapid response following detection of infected cells, and the modulation of other cell types [1]. Together with NK cells, these cell subsets are functionally defined as innate lymphocytes. While innate lymphocytes serve important roles in host resistance to different infections, it remains controversial whether these cells contribute to immunity against *Mycobacterium tuberculosis* (*Mtb*) infection.

Following *Mtb* infection, NK cells become activated and are early and rapid producers of interferon-γ (IFN-γ), a cytokine critical for the activation of macrophages (Mφ) [2],[3]. However, mouse models in which NK cells are defective or are depleted in vivo have failed to show that NK cells are essential for immunity to tuberculosis [3]. Similarly, γδ T cells are frequently activated by a variety of pathogens including *Mtb* [4]. Mice lacking γδ T cells succumb more rapidly than control mice following intravenous challenge with virulent *Mtb*; however, such a difference has not been observed following infection by the aerosol route [5],[6]. Although γδ T cells may not be required for optimum control of bacterial replication following pulmonary infection, γδ T cell deficient mice form disorganized granulomas dominated by foamy Mφ and granulocytes instead of lymphocytes [6]. Similarly, while CD1d-restricted NKT cells rapidly produce large amounts of IFN-γ when activated and play a role in granuloma formation under certain conditions, there is little evidence to support their requirement for optimum immunity against *Mtb* infection, although their pharmacological activation confers a significant survival advantage to susceptible mouse strains [7]–[14].

The mouse model of tuberculosis has been useful in delineating how different cell types contribute to immunity against *Mtb*. Many important components of the human immune response to *Mtb* were first identified or have been successfully modeled in the mouse including the critical role of IL-12, IFN-γ, TNF, and CD4^+^ T cells. CD4^+^ T cells have unambiguously been identified as the most important lymphocyte subset in the mouse for mediating protection. However, the dominant role of CD4^+^ T cells may obscure the contribution of other immune mechanisms. Factors such as inoculum size, *Mtb* strain virulence, and experimental variability limit the dynamic range of the end points measured and reduce the capacity to detect subtle defects in immunity. We established an in vitro model to address whether innate lymphocytes have a role in immunity against *Mtb*. In our model, splenocytes obtained from uninfected mice are cultured with primary Mφ infected with virulent *Mtb*. Under these conditions, splenocytes secrete IFN-γ, stimulate NOS2 upregulation and NO production, and suppress intracellular *Mtb* replication. In this report, we identify the cellular mechanism that mediates this innate effector function against the intracellular human pathogen *Mtb*.

## Results

### Splenocytes from Naïve Mice Are a Source of Innate IFN-γ and Restrict Growth of Mtb

**Figure 1:**
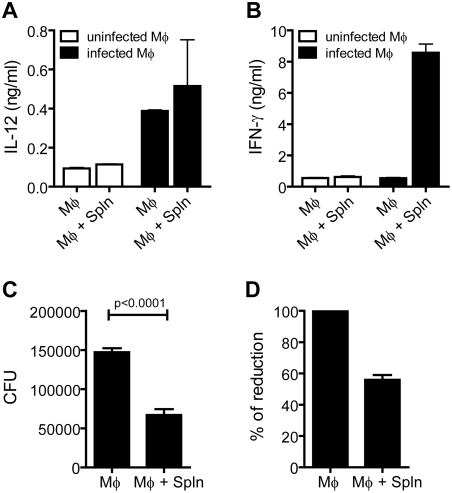
Naïve splenocytes cultured with *Mtb*-infected Mφ produce IFN-γ and suppress bacterial replication. (A) *Mtb*-infected Mφ secrete IL-12. Uninfected (open bars) and *Mtb*-infected (solid bars) were cultured in the presence or absence of splenocytes (Spln). IL-12p40 was measured after 24 hrs. Bars represent the mean+/−SD of triplicate cultures. (B) *Mtb*-infected Mφ stimulate IFN-γ secretion by splenocytes. Uninfected (open bars) and *Mtb*-infected (solid bars) were cultured in the presence or absence of splenocytes. IFN-γ was measured 24 hrs after coculture. The bars represent the mean+/−SD of triplicate cultures. (C) Coculture of splenocytes with infected Mφ leads to reduced *Mtb* replication. *Mtb*-infected Mφ were cultured in the presence or absence of splenocytes from uninfected mice for 3 days and then CFU were determined. Bar represents mean+/−SD from replicate cultures (n = 6). (D) Naïve splenocytes reproducibly suppress intracellular *Mtb* replication. To determine the average CFU reduction, the data from 18 independent experiments were normalized by setting the CFU recovered from the infected Mφ cultured alone to 100%. Bars, mean±SEM.

Elicited peritoneal Mφ were infected with virulent *Mtb* for 2 hrs as described in the Methods. The Mφ were cultured overnight after infection, and the next day splenocytes obtained from uninfected C57BL/6 (B6) mice were added. After 24 hrs, IL-12 was detected in the supernatant of infected macrophages irrespective of whether splenocytes were added (Figure 1A). In contrast, IFN-γ was detected only in the supernatant from cocultures of splenocytes and infected Mφ, but not from cultures of splenocytes and uninfected Mφ, nor from infected Mφ alone (Figure 1B).

Three days after the addition of the splenocytes, the number of bacteria contained in the cultures was determined. Compared to infected Mφ cultured alone, the addition of splenocytes led to a 54.5% reduction of *Mtb* CFU (p<0.0001) (Figure 1C). The precise number of bacteria used to infect the Mφ varied between experiments; therefore, to compare experiments, the data was normalized based on the CFU recovered from infected Mφ 4 days after in vitro infection. This analysis revealed that co-culture of naïve splenocytes with infected Mφ led to a 44.1±3.1% (n = 18 experiments) reduction in mycobacterial growth compared to infected Mφ cultured alone (Figure 1D). Thus, infected Mφ stimulated the production of IFN-γ by naïve splenocytes which correlated with a reduction in the number of *Mtb* recovered from the cultures.

### Suppression of Mycobacterial Growth by Splenocytes Is T Cell–Dependent

**Figure 2:**
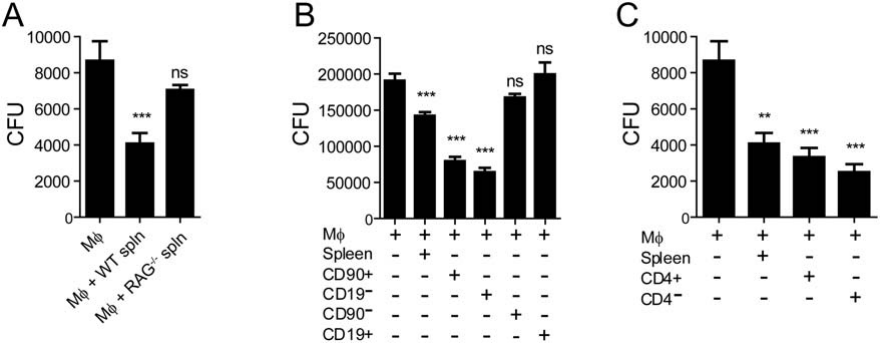
Suppression of *Mtb* replication by splenocytes is T cell–dependent. Statistical testing was done using a one-way ANOVA with Dunnett's post-test comparing each experimental group to infected Mφ cultured alone. Bars, mean±SEM of replicate cultures (n = 6–8). **, ***, p<0.05; ns, not significant. (A) Splenocytes (spln) from RAG*^−/−^* mice are unable to limit bacterial replication. Infected Mφ were cultured alone or with splenocytes from uninfected WT or RAG*^−/−^* mice. Coculture with WT splenocytes but not RAG*^−/−^* splenocytes led to a significant reduction in bacterial CFU after 3 days. (B) T cell enriched cellular fractions have increased anti-mycobacterial activity. Infected Mφ were cultured alone or with cell fractions from WT splenocytes (spleen). CD90 and CD19 immunomagnetic beads were used to enrich or deplete T cells or B cells from splenocytes. (C) Both CD4^+^ and CD4^−^ cells suppress *Mtb* replication. Infected Mφ were cultured alone or with CD4^+^ or CD4^−^ cell fractions obtained from WT splenocytes.

The main cellular source of IFN-γ is NK and T cells. To determine the cellular basis for control of *Mtb* replication, we first determined whether splenocytes from uninfected RAG^−/−^ mice, which lack mature B and T lymphocytes but are enriched for NK cells, could restrict bacterial growth. Compared to splenocytes from uninfected B6 mice, splenocytes from B6 RAG^−/−^ mice were unable to reduce mycobacterial CFU (p = 0.23) (Figure 2A).

The inability of RAG^−/−^ splenocytes to limit *Mtb* growth argues that a B or T lymphocyte subset is required to restrict bacterial replication. To determine which cell type was critical in this process, splenic T cells (CD90^+^) and B cells (CD19^+^) were purified from naïve B6 mice. Purified B cells (CD19^+^), or splenocytes depleted of T cells (CD90-) were unable to control mycobacterial growth (p>0.05, compared to infected Mφ alone) (Figure 2B). In contrast, purified T cells (CD90^+^), or splenocytes enriched in T cells (CD19^−^) very efficiently controlled bacterial growth (p<0.05) (Figure 2B). The increased activity of T cells to control bacterial growth compared to total splenocytes (p<0.05), strongly suggests that suppression of mycobacterial replication is dependent on T cells.

To further characterize the T cell subset that restricts bacterial replication, T cells from spleens of naïve B6 mice were fractionated. We compared the ability of purified CD4^+^ and CD4^−^ cells to reduce CFU. Our results showed that both CD4^+^ and CD4^−^ cells control bacterial growth (Figure 2C). However, the anti-mycobacterial activity of CD4^+^ and CD4^−^ cells was not significantly different. While this result may indicate that either CD4^+^ or CD8^+^ T cells can restrict mycobacterial growth, this effect could be dependent upon NKT cells. Although NKT cells account for only a small fraction of total splenocytes, they can have either a CD4^+^ or CD4^−^8^−^ phenotype, and thus, they would be predicted to be present in both the CD4^+^ and CD4^−^ T cell compartment.

### Recognition of Infected Mφ by CD1d-Restricted iNKT Cells Is Required for the Suppression of Mycobacterial Growth

**Figure 3:**
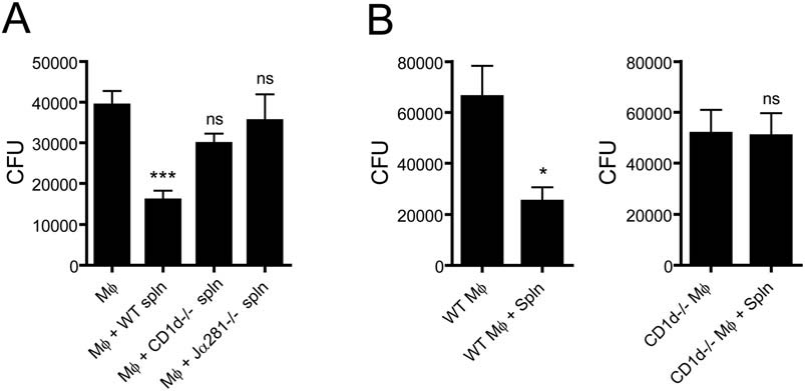
Suppression of bacterial replication requires cognate recognition of *Mtb*-infected Mφ by iNKT cells. Statistical testing was done using a one-way ANOVA with Dunnett's post-test comparing each experimental group to infected Mφ cultured alone. Bars, mean±SEM of replicate cultures (n = 6–8). *, ***, p<0.05; ns, not significant. (A) The presence of iNKT cells is required for the *Mtb* suppressive activity of splenocytes. Infected Mφ were cultured alone or with splenocytes (spln) from WT, CD1d*^−/−^*, or Ja281*^−/−^* mice. Only addition of WT splenocytes led to a significant reduction in CFU. (B) CD1d expression by *Mtb*-infected Mφ is required for the control of bacterial replication. Mφ obtained from WT (left) or CD1d*^−/−^* (right) mice were infected with *Mtb*. Infected Mφ were cocultured with WT splenocytes (spln). Addition of splenocytes to WT Mφ but not CD1d*^−/−^* Mφ led to a significant CFU reduction.

To test the hypothesis that inhibition of mycobacterial growth is dependent on CD1d-restricted NKT cells, we tested whether splenocytes from two different mouse strains that lack NKT cells could reduce *Mtb* CFU. CD1d*^−/−^* mice lack both invariant and diverse CD1d-restricted NKT cells; in contrast, Jα281^−/−^ mice are deficient only in invariant NKT (iNKT) cells. Naïve splenocytes from naïve CD1d*^−/−^*, Jα281^−/−^ or WT mice were cocultured with infected Mφ and bacterial growth was measured 3 days later. Compared to WT splenocytes, both CD1d*^−/−^* and Jα281^−/−^ splenocytes had lost the capacity to control mycobacterial replication (p>0.05 compared to Mφ alone). Thus, the ability of splenocytes to limit mycobacterial growth is dependent upon iNKT cells (Figure 3A)

The requirement for iNKT cells in the control of mycobacterial growth raised the possibility that these cells were being activated by the infected Mφ. Both CD1d-dependent and CD1d-independent mechanisms of iNKT cell activation have been described [15]. Infection can lead to CD1d-dependent iNKT cell activation if there is cognate recognition of microbial antigens, or if the pathogen induces sufficient cytokine production to drive the activation of self-reactive iNKT cells [16]. To determine whether the expression of CD1d by the infected Mφ was required for control of bacterial replication by iNKT cells, Mφ from CD1d*^−/−^* and B6 WT mice were infected in vitro. Naïve splenocytes were added and bacterial growth was measured 3 days later. Naïve splenocytes co-cultured with infected WT Mφ reduced the *Mtb* CFU by 62% (p<0.05) (Figure 3B). In contrast, no CFU reduction was observed when the same splenocytes were cultured with CD1d*^−/−^* Mφ (p, NS). This shows that CD1d expression by the infected Mφ is required for the control of *Mtb* growth by splenocytes. Thus, CD1d-restricted recognition between the infected Mφ and the iNKT cell is required for suppression of bacterial replication.

### Both iNKT Cells and Conventional T Cells Become Activated When Cultured with Mtb-Infected Mφ

**Figure 4:**
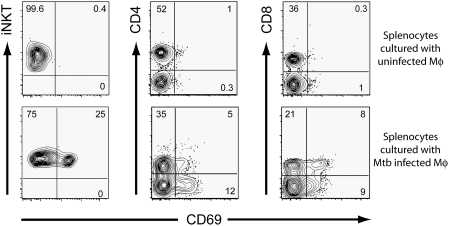
CD69 is upregulated on T cells following culture of splenocytes with *Mtb*-infected Mφ. Splenocytes were cultured with uninfected (top row) or infected (bottom row) Mφ for 24 hrs. Nonadherent cells were recovered and analyzed by flow cytometry. Cells were gated by size and positive staining with CD1d tetramer (left), CD4 (middle), or anti-CD8 (right). CD69 expression was measured as an indication of cell activation.

Activated iNKT cells modulate the activity of other cells including B and T cells, NK cells, granulocytes, and DC. Therefore, while the anti-mycobacterial activity of naïve splenocytes may depend on iNKT cells, iNKT cells may not be sufficient to mediate bacterial growth restriction. To determine what other cell types become activated following culture with *Mtb*-infected Mφ, we measured the expression of the CD69 activation marker 24 hrs after coculture of splenocytes with uninfected or *Mtb*-infected Mφ. CD69 expression remained low on CD4^+^, CD8^+^, and iNKT cells cultured with uninfected Mφ (Figure 4, top row). In contrast, within 24 hrs after culture with infected Mφ, iNKT cells upregulated CD69 expression from 0.4% to 25.2% (Figure 4, bottom row). Similarly, CD69 expression increased on CD4^+^ T cells from 0.9% to 5.2%, and on CD8^+^ T cells from 0.3% to 7.9% (Figure 4). Splenocytes from CD1d*^−/−^* mice were analyzed under these same conditions. Just as with the WT splenocytes, upregulation of CD69 by CD4^+^ and CD8^+^ T cells was observed within 24 hrs after culture with infected Mφ (data not shown). These results show that *Mtb*-infected Mφ stimulate the activation of iNKT cells, CD4^+^ and CD8^+^ T cells. However, the induction of CD69 expression by conventional CD4^+^ and CD8^+^ T cells is CD1d-independent.

### iNKT Cell Effector Function Is Dependent on IL-12 and IL-18 Cytokine-Driven Activation

Following *Mtb* infection, a variety of cytokines are made by infected Mφ, including IL-12 and IL-18, which can drive the antigen-independent production of IFN-γ by T cells. To determine whether IL-12 and IL-18 secreted by infected Mφ are required for iNKT cell activation, antibodies to anti-IL-12p40, anti-IL-18, or both were added to co-cultures of naïve splenocytes and infected Mφ.

**Figure 5:**
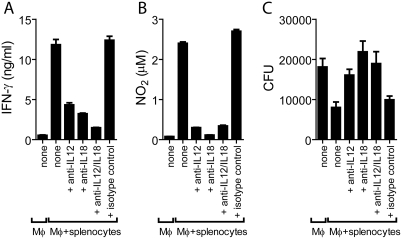
IFN-γ production by innate lymphocytes upregulates NO synthesis and is dependent on IL-12 and IL-18. *Mtb*-infected Mφ were cultured alone or with splenocytes. In addition, some cultures included monoclonal antibodies to IL-12, IL-18, or both, or an irrelevant isotype control antibody. After 24 hrs, IFN-γ (A) and NO_2_ (B) were measured in the culture supernatant, and after 72 hrs the total CFU were determined (C).

Culture of splenocytes with infected Mφ led to the production of γ-interferon (IFN-γ) and nitric oxide (NO) in the culture supernatant (Figure 5A and 5B). These are important mediators of anti-mycobacterial immunity, and IFN-γ is required for optimal induction of inducible nitric oxide synthase (iNOS), which leads to intracellular production of NO, an important effector molecule that ultimately suppresses the growth of *Mtb*. Blockade of IL-12p40 or IL-18 significantly abrogated the secretion of IFN-γ in these cultures (Figure 5A). A predictable consequence was a reduction in NO production (Figure 5B).

In agreement with the reduce secretion of IFN-γ and NO, blockade of IL-12 or IL-18 abrogated control of bacterial replication. Thus, iNKT cell activation and control of the bacterial replication was dependent on both IL-12 p40 and IL-18 (Figure 5C).

### iNKT Cells Are Sufficient To Kill Intracellular Mtb

The results presented above show that the ability of splenocytes to restrict mycobacterial growth is dependent upon iNKT cells. Because iNKT cells have both direct effector and immunoregulatory functions, we next determined whether iNKT cells were sufficient to restrict the growth of *Mtb* or whether this effect was mediated by cross-talk, which activates other cell types to produce IFN-γ. To assess whether invariant NKT cells can directly kill *Mtb*, splenocytes from uninfected B6 mice or short-term iNKT cell lines were cocultured with infected Mφ.

**Figure 6:**
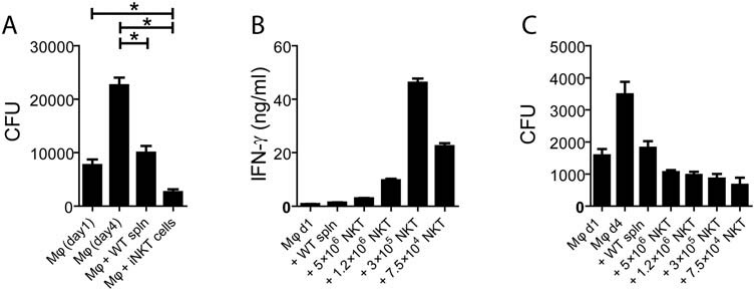
iNKT cells are sufficient to inhibit intracellular bacterial replication. (A) *Mtb*-infected Mφ were cultured alone or with WT splenocytes (spln) or with iNKT cells. Bacterial growth in the *Mtb-*infected Mφ was assessed by determining CFU on day 1 and day 4 post-infection. On day 1, WT splenocytes or the NKT cell line were added to the *Mtb*-infected Mφ, and CFU were determined 72 hrs later on day 4. *, p<0.05, as determined by a one-way ANOVA with Tukey's Multiple Comparison Test. (B) *Mtb-*infected Mφ were cultured alone or with WT splenocytes or with different numbers of NKT cells as indicated. After 24 hours of coculture, IFN-γ was measured in the culture supernatants by ELISA. (C) After 72 hrs, the cultures from (B) were analyzed for the total CFU and compared to *Mtb*-infected Mφ alone on day 1 and day 4. All conditions containing splenocytes or iNKT cells had significantly (p<0.05) fewer CFU compared to *Mtb*-infected Mφ on day 4 post-infection.

On day 1 after infection, 7,663 CFU were recovered after the lysis of *Mtb*-infected Mφ. After three additional days of culture, the CFU increased 3-fold to 22,587. As previously observed, the addition of naive splenocytes on day 1 reduced the number of CFU recovered on day 4 to 9,992 CFU, which represents a 56% reduction compared to infected Mφ alone on day 4 (p<0.05) (Figure 6A). As addition of splenocytes reduced the number of CFU close to, but not below the day 1 value, this reduction represents a suppression of bacterial growth but not killing of *Mtb*.

Addition of purified iNKT cells reduced the CFU by 88%, demonstrating that iNKT cells are sufficient to mediate this anti-bacterial effect (p<0.05, compared to infected Mφ alone, day 4) (Figure 6A). Interestingly, the number of bacteria recovered was lower even than the number of *Mtb* recovered on day 1 (68.4% reduction, p<0.05 compared to infected Mφ alone, day 1) (Figure 4). Thus, not only are iNKT cells necessary and sufficient to restrict intracellular *Mtb* replication, but they have the capacity kill *Mtb*.

Next, we determined whether a physiological number of NKT cells cultured with infected Mφ could have a similar effect. iNKT cells account for 2–5% of splenocytes in B6 mice, which means that 100,000–250,000 iNKT cells are present among the five million splenocytes added to the infected Mφ. Thus, we varied the number of pure iNKT cells from five million to as few as 75,000. Interestingly, fewer iNKT cells led to higher IFN-γ production (Figure 6B). The number of bacteria remaining in the cultures was determined on day 3, and even the presence of 75,000 iNKT cells were able to limit bacterial replication and did so more efficiently than splenocytes (Figure 6C). Again it was observed that after infected Mφ were cultured with purified iNKT cells for 3 days, fewer bacteria remained than were present when the iNKT cells were added (Mφ day 1), suggesting that the iNKT cells induced bacterial killing (Figure 6C).

### iNKT Cells Reduce the Bacterial Burden of Mtb-Infected Mice

**Figure 7:**
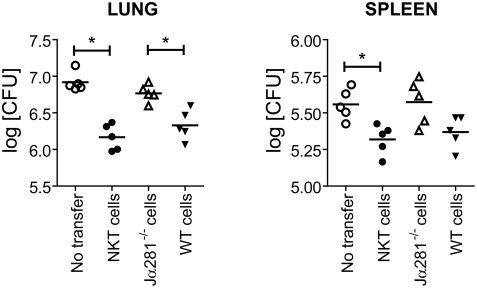
iNKT cells protect mice against *Mtb*. C57BL/6 mice were sublethally irradiated and used as recipients in an adoptive transfer experiment (n = 5/group). As a control, one group did not receive any cells (no transfer). Mice received an iNKT cell line, or splenic CD8^−^CD62L^−^ cells from Jα281*^−/−^* mice or WT C57BL/6 mice. All mice were infected within 24 hrs with *Mtb* by the aerosol route. Three weeks after infection, the bacterial burden in the lung and spleen were determined. Each point represents data from an individual mouse, and the bar represents the mean. The comparison between untransferred mice and mice receiving the NKT cell line was performed twice with similar results. *, p<0.05 by a one-way ANOVA.

Our in vitro results show that *Mtb*-infected Mφ induce iNKT cell activation, which leads to macrophage production of NO, which is associated with the suppression of bacterial growth and perhaps kill *Mtb*. We have previously shown that CD1d*^−/−^* mice are not more susceptible to *Mtb* infection [7]. To reevaluate the potential beneficial role of iNKT cells during *Mtb* infection, NKT cells were transferred into irradiated C57BL/6 mice, which were infected with virulent *Mtb* by the aerosol route. Three weeks later the mice were sacrificed and the CFU in the lung and spleen determined. A significant CFU reduction was observed in both the lung and spleen (Figure 7).

To further assess the protective capacity of iNKT cells, we transferred splenic lymphocytes from uninfected C57BL/6 mice that were enriched for iNKT cells by depleting cells expressing CD8 and CD62L (data not shown). As a control, the same cell population was obtained from Jα281*^−/−^* mice, which lack iNKT cells. The cells obtained from WT mice provided protection to irradiated recipient mice against *Mtb* (Figure 7). In contrast, the cells obtained from Jα281*^−/−^* mice were unable to transfer protection. These data show that primary iNKT cells that had never been activated in vitro are capable of protecting immunocompromised host from *Mtb*.

## Discussion

We find that *Mtb*-infected Mφ stimulate IFN-γ production by splenocytes. Since the splenocytes are obtained from uninfected mice that have no previous exposure to *Mtb* and thus are not “immune” with respect to *Mtb* antigens, their IFN-γ production must represent activation of an innate pathway. Surprisingly, the production of IFN-γ is dependent on iNKT cells, a minor subset of T cells that is present in the spleen at a frequency of ∼1%. The biology of iNKT cells has been well described during the past decade: they are CD1d-restricted T cells that express an invariant TCRα, rapidly produce cytokines when activated, and modulate a variety of immune phenomenon [17]–[19].

How do *Mtb*-infected Mφ activate iNKT cells? There are several models of how CD1d-restricted T cells become activated. Presentation of microbial lipid antigen has been described for certain infectious agents [20]–[23], and although *Mtb* antigens are known for group 1 CD1, and it's possible that CD1d presents *Mtb* ligands, our study does not directly address this possibility. In our model, iNKT cell effector function is dependent upon CD1d expression by the infected Mφ, indicating that CD1d recognition is required. Whether a microbial antigen or an endogenous self lipid antigen is recognized is not certain. Both IL-12p40 and IL-18 are required for the iNKT cell effector function we observe. Thus, our data is consistent with iNKT cell recognition of a self or microbial antigen and costimulation by IL-12 and IL-18, two cytokines which are produced by *Mtb*-infected Mφ. Not only does IL-12p40 and IL-12p35 form the heterodimeric cytokine IL-12p70, but IL-12p40 also pairs with IL-23p19, to form the cytokine IL-23. Since IL-23 is induced by mycobacterial infection of Mφ [24],[25], an alternate interpretation of our data is that IL-23 has a role in iNKT cell activation. Following iNKT cell activation, IFN-γ is produced early and stimulates infected Mφ to produce iNOS. This leads to the accumulation of NO, which is associated with suppression of bacterial growth.

Many studies have described how iNKT cells contribute to microbial immunity (reviewed in [15],[26],[27]). iNKT cells are frequently activated early during infection and secrete IFN-γ, which can have an immunomodulatory role. The use of CD1d*^−/−^* and Jα281*^−/−^* mice has clearly revealed that iNKT cells can either exacerbate or ameliorate the outcome of infection. In addition, pharmacological activation of iNKT cells with the synthetic ligand αGalCer often enhances host resistance to infection. iNKT cell use several mechanisms to modify host immunity. These include induction of DC maturation, secondary activation of effector cells (NK cells) or recruitment of inflammatory cells to the site of infection (PMNs) [28]–[32]. However, this is the first example of iNKT cells having a direct effector function against a microbial pathogen.

Whether CD1d-restricted T cells are required for immunity against *Mtb* has been partially addressed by several studies. Both CD1d*^−/−^* and Jα281*^−/−^* mice have been infected with virulent *Mtb* by both the IV and aerosol route. All studies to date have found that the absence of CD1d-restricted T cells do not alter the outcome to tuberculosis infection in the mouse model [7]–[11]. Nevertheless, treatment of mice with αGalCer, which specifically activates iNKT cells, significantly prolongs the survival of infected mice [14]. In fact, a single dose of αGalCer given the day after infection prolongs the survival of mice, an endpoint quite remote from its administration. Thus, it may be that iNKT cells are not activated optimally in mycobacterial infection but they can alter the long-term outcome of infection when pharmacologically activated. A physiological role for this T cell subset early during infection has been suggested by Szalay et al, who found that treatment of *Mtb*-infected mice with anti-CD1 mAb diminished control of bacterial replication early after IV infection and correlated with reduced IFN-γ and TNF production by *Mtb* antigen stimulated splenocytes [33]. While another study found that anti-CD1d mAb treatment did not worsen the pulmonary *Mtb* burden following low dose aerosol infection, early time points were not examined [8]. Our results are consistent with an early innate role for iNKT cells in controlling *Mtb* infection.

Why are CD1d^−/−^ mice, which lack the iNKT cell subset, no more susceptible than WT mice to *Mtb* infection? There may be insufficient numbers of resident iNKT cells in the lung to mediate an anti-mycobacterial effect early during the course of infection. Although we observed that only a few iNKT cells are needed to suppress *Mtb* replication, the statistical probability of finding an iNKT cell and an infected Mφ in the same alveoli is low. In contrast, once infection is established, iNKT cells are recruited to the lung and Mφ upregulate cell surface CD1d [34]; however, this is coincident with the appearance of conventional T cells in the lung, which may overshadow the contribution of CD1d-restricted T cells. If IFN-γ production is the key function of CD1d-restricted T cells, CD1d and Jα281 ko mice may lack a phenotype because this function can be mediated by other cell types. Cytokines such as IL-12 and IL-18 can drive IFN-γ production by γδ T cells, NK cells, and memory CD8 T cells independently of cognate antigen recognition. Finally, an intriguing observation is the development of iNKT cell anergy following BCG infection [35] and other bacterial infections [36]. If iNKT cell anergy were to develop during *Mtb* infection, then iNKT cells would appear to make little or no contribution to immunity against *Mtb* infection. We speculate that the basis of the protective effect of αGalCer lies in its ability to activate iNKT cells before anergy develops.

We established an in vitro model to study innate cellular mechanisms of immunity against intracellular *Mtb*. We find that macrophages infected with virulent *Mtb* stimulate the IFN-γ production by non-immune splenocytes, which inhibits bacterial replication. These cellular events are entirely dependent on iNKT cells forcing us to re-examine the role of CD1d-restricted iNKT cells in vivo. Using a primary iNKT cell line, we show that iNKT cells provide protection against low dose aerosol *Mtb* infection. These data demonstrate that iNKT cells have a physiological role in limiting *Mtb* infection by acting directly as effector T cells. These findings provide a rationale for understanding how immunomodulation of CD1d-restricted T cells during *Mtb* infection can have a role in the adjuvant therapy of tuberculosis.

## Methods

### Mice

C57BL/6 (B6) mice were obtained from The Jackson Laboratory. CD1d knockout (CD1d^−/−^) mice on the B6 background were kindly provided by Mark Exley [37]. Jα281 knockout (Jα281^−/−^) mice were provided by M. Taniguchi (Riken, Japan) [38]. Female mice, 7–8 weeks of age, were used in all experiments. Mice were bred and maintained under specific pathogen-free conditions and were used in a protocol approved by the Dana Farber Cancer Institute.

### Antibodies and CD1d-PBS57 Tetramers

The following FITC-, PE-, PECy7-, allophycocyanin-, and PerCP-conjugated anti-mouse mAbs were purchased from BD Pharmingen: anti-CD8 (PerCP), anti-CD3 (APC), anti-CD69 (PECy7) and anti-CD4 (FITC). Anti-mouse IL-18 was purchased from MBL, anti-mouse IL-12/IL-23 p40, mouse IgG1 and IgG2A isotype controls were purchased from RD systems. CD1d-PBS57 and control PE-conjugated tetramers were provided by the National Institute of Allergy and Infectious Diseases Tetramer Facility (Emory University Vaccine Center, Atlanta, GA).

### Peritoneal Mφ and In Vitro Culture

Peritoneal exudate cells were harvested by intraperitoneal lavage 4 days after intraperitoneal injection of sterile 3% thioglycolate medium [34]. Mφ were purified in a two steps protocol: B cells were depleted using anti-CD19-microbeads followed by positive selection of Mφ with CD11b-microbeads (Miltenyi Biotech). The purified cells were >95% F4/80^+^ CD11b^+^, as determined by flow cytometry. Purified Mφ (1×10^6^) were seeded into 24-well plates in complete RPMI 1640 medium (Invitrogen Life Technologies) supplemented with 10% fetal calf serum (HyClone), penicillin/streptomycin, L-glutamine, sodium-pyruvate, 2-ME, nonessential amino acids, essential amino acids, and HEPES buffer (all from Gibco). Recombinant mouse IFN-γ (US Biological) was used at the concentrations indicated.

### Bacteria and In vitro Infections

Virulent *Mtb* (H37Rv) was grown to mid-log phase in Middlebrook 7H9 medium containing 10% albumin/dextrose/catalase enrichment (BD Biosciences). Bacteria were opsonized for 2 minutes using RPMI 1640 medium with 2% human serum (Gemini Bio-Products), 10% FBS, and 0.05% Tween 80 and then washed twice with complete medium without antibiotics. Bacteria were passed through a 5-µm syringe filter (Millipore), counted in a Petroff-Hausser chamber and added to enriched Mφ at different multiplicity of infection (MOI) as indicated. The length on infection was 2 hr for all experiments. Infected Mφ were cultured overnight before the addition of splenocytes or purified cell subsets (see below). In some experiments, infected Mφ were cultured overnight in the presence of rIFN-γ (2.5 units/ml). The next day all wells were washed twice with RPMI medium without antibiotics.

### Splenocytes and Cell Enrichment

Spleens were aseptically removed and mechanically homogenized with a 3-ml syringe plunger. Erythrocytes were lysed with RBC lysis buffer (1 mM KHCO3, 0.15 M NaCl and 0.1 mM sodium-EDTA [pH 7.3]). Cells were washed and viability determined using trypan blue. Splenocytes were resuspended in complete RPMI 1640 medium and unless otherwise specified, 5×10^6^ splenocytes/well were added to cultures of infected Mφ in 24-well plates. T cells (Thy1^+^), B cells (CD19^+^) and CD4^+^ T cells were enriched using microbeads (Miltenyi Biotec, Auburn, CA). Enriched NKT cells were prepared by depleting CD8^+^ and CD62L^+^ cells from splenocytes using microbeads, and contained 12-15% tetramer^+^ T cells.

### NKT Cell Line

T cells were negatively isolated from splenocytes of Vα14-Jα281 Tg mice by using the Pan T cell isolation kit (Mylteni Biotec). NKT cells staining with the APC labeled mouse CD1d tetramer loaded with PBS57 (provided by the NIAID Tetramer Facility) were sorted by using anti-APC MicroBeads (Mylteni Biotec). Bone marrow-derived dendritic cells (BM-DC) were grown from bone marrow progenitors by culture for 6 days in the presence of granulocyte-monocyte colony stimulating factor (10 ng/ml) and IL-4 (1 ng/ml) (R&D systems) in complete RPMI medium (RPMI supplemented with L-glutamine and penicillin/streptomycin; Life Technologies) containing 10% FBS (Hylone Laboratories). BM-DCs were pulsed with αGalCer at 100 ng/ml for 24h at 37°C and then irradiated at 3400 rad. 2×10^6^ NKT cells were cultured with 2×10^5^ αGalCer-pulsed BM-DCs per well in 24 well plates in complete RPMI 1640 medium. 3–5 days later, mIL-2 (10 U/ml) (R&D Systems) and mIL-7 (10 ng/ml) (PeproTech) were added in the medium. NKT cell lines were re-stimulated with αGalCer-loaded BM-DCs every 2–3 weeks. These cells were used for both in vitro and in vivo experiments.

### Blocking Experiments

Cytokine function was blocked by addition of azide-free, low-endotoxin anti-mouse cytokine-specific mAbs. Infected Mφ were co-cultured with naive splenocytes at a ratio of 5:1 in the presence or absence of antibodies specific for IL-12 (10 µg/ml, clone C17.8, R & D) and IL-18 (10 µg/ml, clone 93-10C, MBL) or isotype-matched control mAb. On day 4 after the infection supernatants and cells were recovered to analyze IFN-γ and NO production, and bacterial growth.

### CFU Determination

Bacterial growth was quantified 24 hrs after Mφ infection (hereafter referred to as day 1) and 72 hr after coculture with naive splenocytes or splenocyte cell fractions (day 4). After removing the culture supernatant, cells were lysed by adding distilled water for 3 min and plating 10-fold dilutions on Middlebrook 7H11 agar plates. In some experiments, the number of CFU contained in the supernatant was also determined. The number of bacteria contained in the supernatant was <10% of the cell associated CFU, and this was true whether or not splenocytes or other cell fractions were added to the infected Mφ. The number of colonies was counted 3 weeks after incubation at 37°C in a humidified CO_2_ atmosphere.

### Cytokine Detection

Culture supernatants were assayed for IFN-γ and IL-12p40 by standard sandwich ELISA. All Ab pairs and standards were purchased from BD PharMingen, and ELISAs were done in accordance with the manufacturer's instructions. Samples were read at 405 nm on SoftMax Pro ELISA analysis software (Molecular Devices). IFN-γ and IL-12p40 were quantified by comparison with the appropriate recombinant standard (purchased from BD PharMingen).

### NO Production

Supernatants were harvested after 24 hrs of coculture and NO production was measured using the Griess reaction. Briefly, 100 µl of culture supernatants were incubated with an equal volume of commercial Griess reagent (Sigma-Aldrich) for 5 min at room temperature, and the absorbance was measured at 490 nm. Serially diluted NaNO_2_ of known concentration was used as a standard to calculate the amount of NO_2_ found in the supernatant [39].

### Flow Cytometry

Changes in the phenotype of cells 24 hrs after coculture with infected Mφ was determined by multiparametric flow cytometry. Recovered cells were resuspended in FACS buffer (2% FCS, 2 mM sodium azide in PBS). To inhibit nonspecific staining, cells were incubated with 25 µg/ml of anti-FcγRII/III Ab (2.4G2) for 10 min at 4°C, washed, and then stained with fluorochrome-conjugated isotype-matched control IgG or Abs specific for mouse cellular markers. Cells were also stained with CD1d-PBS57 tetramer in FACS buffer for 15–20 min at 4°C, and then washed and fixed in 1% paraformaldehyde overnight. Data was collected using a FACSCanto (BD Biosciences) and analyzed with FlowJo (Tree Star).

### NKT Cell Adoptive Transfer into *Mtb*-Infected Recipient Mice

An adoptive transfer system was used to analyze the ability of NKT cells to mediate protection during pulmonary tuberculosis infection. B6 mice were sublethally irradiated with 600rads from a cesium-137 source. The next day, the mice were infected with virulent *Mtb* (Erdman strain) via the aerosol route as described previously [40]. Invariant NKT cells (5×10^6^/mouse) or enriched splenic NKT cells (10×10^6^/mouse) were injected via the tail vein into the infected B6 mice. Three weeks after infection and adoptive transfer, the mice were euthanized and their left lung and spleen were aseptically removed and individually homogenized in 0.9% NaCl-0.02% Tween 80 with a Mini-Bead-Beater-8 (BioSpec Products, Bartlesville, OK). Viable bacteria were enumerated by plating 10-fold serial dilutions of organ homogenates onto 7H11 agar plates (Remel). Colonies were counted after 3 weeks of incubation at 37°C.

### Statistical Analysis

Data are shown as mean±standard deviation (SD). Unpaired student's test was used to compare two groups and one way analysis of variance (ANOVA) when more than two groups were compared. A level of p <0.05 was accepted as statistically significant. For the in vivo experiments, CFU were log_10_ transformed before statistical testing using the non-parametric Mann-Whitney test. Analysis was performed using Prism 5.0 software (GraphPad Software, Inc., San Diego, CA).
